# How to Manage Disasters while Considering Responsibility to Protect Theory?

**DOI:** 10.30476/BEAT.2021.88343.1202

**Published:** 2021-07

**Authors:** Hasan Esfandiar, Mahmoudreza Peyravi, Milad Ahmadi Marzaleh, Hojatolah Najafi

**Affiliations:** 1 *Director General International Communications, Iranian Red Crescent Society, Tehran, Iran*; 2 *Department of Health in Disasters and Emergencies, Health Human Resources Research Center, School of Management and Medical Informatics, Shiraz University of Medical Sciences, Shiraz, Iran*; 3 *Helal-Iran Institute, Red Crescent Society of the Islamic Republic of Iran, Tehran; Research Center for Health Management in Mass Gathering, Red Crescent society of the Islamic Republic of Iran, Tehran; Student Research Committee, Department of Health in Disasters and Emergencies, Health Human Resources Research Center, School of Management and Medical Informatics, Shiraz University of Medical Sciences, Shiraz, Iran MPH of Health Policy, Health Policy Research Center, Institute of Health, Shiraz University of Medical Sciences, Fars, Iran*; 4 *Student Research Committee, Department of Health in Disasters and Emergencies, Health Human Resources Research Center, School of Management and Medical Informatics, Shiraz University of Medical Sciences, Shiraz, Iran*

**Dear Editor**

Disasters are an integral part of human life, which have been grown increasingly in recent years. Disasters are divided into human-made and natural categories [1]. In this study, two cases are considered. One is deliberate human-made disasters in such conditions that human crimes or vicious crimes occur in countries, and the government is the criminal factor or is not able to control the crimes. The other case is natural disasters in which people are hurt and suffer from death and diseases, and although the host country is not able to be responsive which it refuses to accept international grants. 

In this regard, the global community is faced with several challenges, namely sovereignty right of states, ethical and philanthropic interventions, flexibility in the current rules, and rule of selecting and taking. Sovereignty right has been defined as follows: “No state is subject of other states, and the state itself has complete and monopolized power in its judicial jurisdiction framework [2]. Although increasing the importance of human rights led to this belief, the states should not be premised to violate citizens’ human rights under the shadow of sovereignty right [3]. On the other hand, conscience and humanity affect nations and states in such conditions and force them to have unilateral philanthropic interventions. Therefore, armed interventions have been seen in Somalia and Kosovo by international communities. Thus, there is a challenge between sovereignty right and philanthropic interventions. Another challenge is that all countries are not forced to intervene and are not responsible in this regard. Also, there are apparent cases of violence against humanity in conditions such as Rwanda, and Sudan, in which the global community did not tend to have philanthropic interventions. Therefore, the existing rules make a flexible environment in which international communities themselves select whether they should have an armed intervention or not and a comprehensive and definitive framework was needed to eliminate these challenges [4]. 

The so-called challenges led to the formation of the Responsibility to Protect (R2P or RtoP) doctrine, which was propounded in 2001 and confirmed by the General Assembly of the United Nations (UN) in 2005. In cases that a state is not able or does not tend to protect its people against crimes such as genocide, war crimes, ethnic cleansing, and offenses against humanity, other states accept the protective responsibility of those people. RtoP involves preventive, response, and restoration domain [2, 5]. 

The second scenario is related to natural disasters and the host’s state inability to respond and dismiss international aids despite extensive human harm and death. How should the international society be responsive in these cases?

For example, the Nargis cyclone rolled up some southern regions of Myanmar during the 2^nd^ and 3^rd^ days of May in 2008. Fifty thousand to seventy thousand cases are seemed as optimistic dead based on the first estimation. At last, one hundred and forty thousand cases were killed and missed. Other grants were gathered, and they were transmitted to the disaster site, but the governed military alliance state did not accept the grant. Hence, the rate of death increased while the transmitted grants were useless in harbors. This question is repeated that what right or duty other nations and states have in such conditions [6].

RtoP has not regarded natural disasters, so, armed interference has not been applied in natural disaster cases. Therefore, the international society should imagine itself in a condition such as the Myanmar storm, and present a solution for responding to these conditions by using protective responsibility frameworks [7]. However, the sovereignty’s right challenge appears in this domain because aids must be based on the host state’s satisfaction. The International Committee of Intervention and Sovereignty Right has mentioned the term *responsibility to protect* instead of *protective right* to protect people in states’ territory in order to solve this problem. States are responsible for protecting their people against disasters. Natural disasters cause numerous problems for people and accompanied by lots of deaths. If a state cannot meet its people’s requirements and refuses international aiding delivery, it does not observe human rights and is responsible for suffering, torment, and human rights crime, so other states should have the protective responsibility. Therefore, RtoP can be applied to solve the challenge between the sovereignty right and philanthropic interventions and can be generalized in natural disasters as well [3].

Also, the responsible authority is faced with another problem about the application of RtoP in natural disasters. There is no law that can force states to protect, and protective responsibility is justified based on the existing international treaties and rules. Therefore, since there is no compulsory law, this problem must be justified based on the existing international rules [3].

Finally, besides some advantages, RtoP has some weaknesses. It is like a double-edged sword that may be abused for justifying unilateral interventions in civil affairs of countries considering the subject of Libya. Another weakness of RtoP is its close and false relationship with philanthropic interventions, which can lead to the misinterpretation of this subject by superpowers and lead them to make legitimate interferences in other countries’ affairs. Overall, this doctrine has some limitations, and some critics must be averted in order to promote and transform it into a custom [2].

The main purpose of the RtoP doctrine is philanthropic measures and reduce the community suffering and death, which happens in the case of human-made disasters. As intentional human disasters can lead to human rights infringement, a protection lack for the suffering people from natural disasters will have similar results. Hence, the RtoP doctrine that considers philanthropic protections beside the country’s sovereignty right can be applied in the case of natural disasters, as well. Dual usages of this rule have been observed in different places, unfortunately. Therefore, the international community needs further development of this concept and bounding states in its correct enforcement.
